# Research on the income and expenditure forecast of long-term care insurance fund in Shihezi City based on community care

**DOI:** 10.3389/fpubh.2024.1329155

**Published:** 2024-05-13

**Authors:** Xu Yang, Penghua Zuo, Huiling Xie

**Affiliations:** ^1^Department of Health Economics, School of Public Health, Xinjiang Medical University, Urumqi, China; ^2^Department of Health Management, School of Medical and Health Management, Tongji Medical College, Huazhong University of Science and Technology, Wuhan, China

**Keywords:** long-term care, income, expenditure, funding standards, prediction

## Abstract

**Objective:**

This study forecasts the income and expenditures of the long-term care insurance fund, provides a basis for formulating the raising standard of the long-term care insurance fund, and explores the measures to improve the pilot work of long-term care insurance.

**Methods:**

By using the exponential smoothing and ARIMA models to forecast the income and expenditure of the old-age care insurance fund in 2022, the problems existing in the operation of the long-term care insurance fund are discussed.

**Results:**

In 2022, the income of the old-age insurance fund was 28.8934 million yuan, and the fund compensation expenditure was 28.4070 million yuan, with a slight balance of the fund. The highest relative errors of income and expenditure forecast models are −2.03% and − 2.76%, respectively. According to the results of fund expenditure, the annual financing standard should be 132.93 yuan/person, and the individual financing standard should be 66.47 yuan/person.

**Conclusion:**

Through the integration of personal payment, welfare, sports lottery public welfare income, social donations, and other ways, we can gradually establish a multi-channel risk-sharing financing. We will appropriately raise the standard for individual financing and the annual contribution standard for individuals from 50 yuan to 66.47 yuan. This will promote sustainable development of long-term insurance system.

## 1 Background

With the development of economic globalization, the gradual improvement of residents’ quality of life, and the advancement of modern medical technology, the mortality rate of populations in various countries has greatly decreased, and the life expectancy of the population has been continuously extended (1). Research shows that, over the past 70 years, the average life expectancy of the world population has increased by nearly 20 years (2). However, this is accompanied by a decline in physical function and self-care ability caused by age, leading to an increase in disabled and semi-disabled older adult people worldwide, and a large-scale increase in long-term care needs (3, 4). Long-term care services refer to the services provided to individuals who require ongoing assistance and care due to aging, illness, or disability. These services typically involve daily activities such as eating, dressing, and bathing. Long-term care services can be provided in various settings, including homes, communities, nursing homes, and specialized facilities such as rehabilitation centers (5). However, China relies relatively less on older adult care institutions, and there is also a shortage of professional caregivers. Additionally, with the large number of single-child families in China, relying on the traditional model of informal care provided by families is unsustainable (6).

In China, community care has been receiving increasing attention and support. Community care services across various regions in China include multiple groups such as older adult individuals, persons with disabilities, children, and impoverished families. Through community health service centers and township health clinics, residents are provided with comprehensive care and support including medical treatment, psychological support, rehabilitation therapy, health education, and other services. Currently, more than 25 million older adult people in China require long-term care. One study estimates that approximately 70% of individuals aged 65 years and above will need some level of long-term care during their remaining years, with 20% of them requiring long-term care for 5 years or longer (7). It is noted that the financial resilience of older adult individuals in our country is extremely limited, and long-term care for the older adult imposes economic and caregiving burdens on both individuals and their families. There is an urgent need to establish a system that alleviates the financial burden on families with disabled older adult individuals and improves their quality of life.

When analyzing the financing of long-term care globally, it is evident that one-third is from taxation and out-of-pocket payments, while another third is secured through social security systems, with models such as the pay-as-you-go social health insurance system implemented in countries such as Japan and Germany (8). This means that today’s working generation funds today’s beneficiaries, redistributing income from current workers to beneficiaries as people accumulate assets for their own future risks. In the pay-as-you-go system, since insurance funds mainly rely on contributions from current workers, the model for sharing and distributing future long-term care risks lacks clarity. This can easily lead to instability in the system or inability to meet the demands (9). However, research shows that implementing a public long-term care insurance system is still beneficial, as insurance premiums are more accepted by the population than higher taxes (10). Although the social long-term care (LTC) insurance model is becoming increasingly popular, to date, only five high-income countries (Germany, the Netherlands, Luxembourg, Japan, and South Korea) have adopted this model. There is little evidence regarding whether this model can achieve similar goals in low- and middle-income countries, where limited income from public funds may restrict insurance benefit programs (11).

Long-term care insurance originated in the Netherlands during the 1960s. It refers to a form of health insurance that compensates for various expenses paid by insured individuals who have lost all or part of their bodily functions due to aging, chronic diseases, accidental disabilities, and other such conditions, rendering them unable to take care of themselves (12). As a result, they require long-term rehabilitation and supportive care from others at home or in a nursing home. With the intensification of population aging worldwide, an increasing number of countries are starting to establish long-term care insurance systems (13). Research has shown that the establishment of long-term care insurance not only effectively addresses the long-term care needs of the older adult, chronic disease patients, and disabled individuals but also improves the health status of those insured. It reduces the economic burden on both the government and individuals, promotes the development of the nursing industry, and increases employment opportunities (4, 14, 15). The experiences and practices of various countries have proven that long-term care insurance (LTCI) is one of the most effective policy tools for addressing the risks of older adult care. Given the rapidly increasing older adult population, rising medical expenses, and weakening family support structures, it is crucial to establish an LTCI system suitable for China’s national conditions.

Compared to other countries, the development of China’s long-term care insurance system has been relatively slow (16). Since the 1970s, China’s population structure has shown a trend toward an inverted pyramid (17, 18). In 2000, China had officially entered a population-aging society, with a large population base, rapid aging, and aging development (19). At the same time, with the transformation of the disease spectrum, chronic non-communicable diseases have become the main factor affecting the health of the population, with over 60% of the older adult being in a state of illness in their later years, resulting in an increasing number of disabled people in China (20, 21). In 2006, the China Insurance Regulatory Commission issued the “Health Insurance Management Measures,” which listed nursing insurance as one of the four major types of health insurance (22). In the same year, the decision on comprehensively strengthening the coordination of population and family planning work to solve population problems pointed out that it is encouraged to build older adult care service institutions, develop the aging industry, and explore the establishment of social service systems such as long-term care insurance for the older adult (23). In 2012, Qingdao became the first pilot city in China to implement a long-term care insurance system (24). In 2016, the Ministry of Human Resources and Social Security of the People’s Republic of China issued the “Guiding Opinions on Pilot Implementation of the Long Term Care Insurance System” (hereinafter referred to as the “Pilot Opinions”), selecting 15 cities as pilot cities to carry out pilot work on long-term care insurance nationwide (25). In 2020, the National Medical Insurance Bureau and the Ministry of Finance jointly issued the “Guiding Opinions on Expanding the Pilot of Long Term Care Insurance System,” which updated and improved the institutional framework, policy standards, and operational mechanisms and added 14 more pilot cities for long-term care insurance.

China has adopted a mandatory social long-term care insurance system (26). (1) This system focuses on addressing the basic living and related care costs of severely disabled individuals. It mainly targets urban employees who are already covered by basic medical insurance. Some cities, such as Changchun, Jingmen, and Nantong, have expanded their coverage to encompass both urban and rural residents under basic medical insurance. Except for Shanghai and Guangzhou, most pilot cities do not have age requirements for insured persons (27, 28), (2) Most pilot cities rely on multi-channel fundraising approach under the medical insurance pooling fund, including financial subsidies, unit and individual contributions, and welfare lottery public welfare funds, among other sources, to raise long-term care insurance funds. Only Shijingshan District of Beijing is completely independent of the medical insurance fund fundraising (29), (3) Long-term care services mainly include care in medical institutions, care in older adult care facilities, and home care (including self-care and care provided at home), (4) The nursing service mainly includes two aspects: basic daily care and medical care. Some cities, such as Jingmen and Ningbo, also include psychological comfort and end-of-life care in their service scope, (5) The payment for long-term care insurance generally varies based on the type of nursing services provided, with a payment ratio of mostly over 50%. Most cities have set a maximum payment limit, and some cities have also designed payment conditions that are “bundled” with the payment period of medical insurance, and (6) Long-term care insurance is handled and managed by social medical insurance institutions in various regions. It is undertaken by commercial insurance companies through government purchase services. The undertaking institutions are responsible for disability level assessments, cost verification, settlement and payment, and other businesses.

According to the 2018 Statistical Bulletin on the Development of Civil Affairs by the Ministry of Civil Affairs, by the end of 2018, China’s long-term care insurance coverage exceeded 50 million people, indicating that the popularization scope of China’s long-term care insurance system is gradually expanding (30). Research has shown that, in the pilot implementation process of long-term care insurance, it not only reduces the care and economic burden on families of disabled individuals but also improves their quality of life. Furthermore, it promotes the development of the nursing industry. However, there are also problems such as narrow insurance coverage, unclear fund sharing, and low fund payment levels (31).

Shihezi City, as the first batch of China, Xinjiang’s first long-term care insurance pilot city, officially started the pilot work of long-term care insurance in July 2017. In 2020, the percentage of older adult people aged 60 years and above in Shihezi City reached 22.51%, far exceeding the national average (18.70%) for the same period. However, the *per capita* disposable income of residents (35,000 yuan) is significantly lower than the national average (43,000 yuan). It is particularly important to ensure the sustainable development of long-term care insurance in the city, meeting the long-term care needs of its residents to the greatest extent possible while reducing their economic burden. This study aimed to provide a basis for the formulation of standards for the fundraising for the long-term care insurance fund by forecasting the reimbursement of the long-term care insurance fund and exploring measures to improve the pilot work of long-term insurance.

## 2 Data sources and measurements

### 2.1 Data sources

1. We collected demographic and socioeconomic development data of Shihezi City from 2017 to 2020 using sources such as the Xinjiang Production and Construction Group Statistical Yearbook and the Statistical Communiqué of National Economic and Social Development of Shihezi City.
2. We collected data from 2014 to 2021 on basic medical insurance for urban employees and urban and rural residents from statistical forms provided by the Shihezi City Medical Security Bureau.
3. We collected data from statistical forms from July 2017 to September 2021 pertaining to the long-term care program and beneficiary information database provided by the Shihezi City Long-Term Care Insurance Coinsurance Office.

## 3 Methods

### 3.1 Exponential smoothing model

The exponential smoothing model was first proposed by Brown, who believed that the trends in the time sequence were stable or regular and could be reasonably postponed in a smoothing manner. For the three circumstances of the time sequence (randomness, tendency, and seasonality), the exponential smoothing model has corresponding methods to predict: Simple index smoothing methods respond to relatively smooth situations, Holt’s dual parameter method responds to trends, and the Holt-Went model responds to seasonal tendencies (32). Regardless of the smoothing method, two parameter values (initial value S0 and smoothing coefficient alpha) are involved. If the sample amount is <10, then the initial amount S0 takes the average of the first 3 periods. If 10 ≤ sample volume is ≤20, then the original value S0 takes the first 2 periods of the average, and if the sampling amount is >20, the initial number S0 takes the average of 1 period (33). If the data fluctuation is significant, an alpha value of approximately 0.6–0.8 should be selected; contrarily, if the data fluctuation is minimal, an alpha value of approximately 0.1–0.5 should be selected. The root mean square error (RMSE) was used to determine the best alpha value to get the best prediction effect (34).

This study used the data on the number of participants covered by basic medical insurance in Shihezi City from 2014 to 2021, and it utilized Excel to build a quadratic exponential smoothing model (Holt’s two-parameter method) to forecast of the number of individuals covered by basic medical insurance. Furthermore, this can be combined with the current long-term care insurance funding standards to forecast long-term care insurance funding revenue of 2022.

### 3.2 Autoregressive integrated moving average (ARIMA) model

The ARIMA model was originally a time series prediction method proposed by American statisticians, Box and Jenkings, in the early 1970s. Therefore, it is also called the Box-Jenkings model (35). The model can be summarized as ARIMA (p, d, q), and p represents the autoregressive order, d represents a sequence that makes the sequence stable by several layers of differentiation, and q represents an moving average order. The steps for developing an ARIMA model are as follows: ① Collecting the time series data. ② Preprocessing time series data, which includes conducting stationarity and white noise tests. The model can only be established using the stationarity white noise series. ③ Model identification. ④ Determination of model order. ⑤ Prediction and model fitting (36).

This study used data of Shihezi City Long-Term Care Insurance Fund compensation expenditure data from August 2017 to September 2021. SPSS 22.0 was used for constructing the ARIMA model to forecast the long-term care insurance fund compensation expenditure.

## 4 Results

Characteristics of the resident population in Shihezi City from 2017 to 2020.

From 2017 to 2020, the total population of Shihezi City has been slowly decreasing with an average annual growth rate of −0.05%. During this period, the proportion of female individuals has been increasing at a rate of 0.12% annually, while the proportion of male individuals has been slowly declining at a rate of −0.12% per year. The proportion of rural population has been decreasing each year with an average growth rate of −7.22%, while the proportion of urban population has been gradually increasing with an average growth rate of 1.97% annually. When divided into age groups, except for the population aged 60 years and above, whose proportion has been increasing year by year, the proportions of the populations aged 0–17 years, 18–34 years, and 35–59 years have all shown negative growth trends. Over the span of 4 years, the birth rate, death rate, and natural population growth rate have all been steadily decreasing. Among these, the natural population growth rate has been declining at the fastest pace with an average annual growth rate of −52.78% (Table 1).

**Table 1:** Demographic data of Shihezi City from 2017 to 2020 (%).

Characteristics	Year 2017	Year 2018	Year 2019	Year 2020	Average annual growth rate(%)
Total population (10,000)	59.25	59.30	58.90	59.16	−0.05
Sex ratio					
Male	49.77	49.65	49.62	49.59	−0.12
Female	50.23	50.35	50.38	50.41	0.12
Proportion of household registration					
Urban	77.00	80.23	81.56	81.63	1.97
Rural	23.00	19.77	18.44	18.37	−7.22
Age ratio(years)					
0~	12.78	12.79	12.66	12.52	−0.68
18~	20.12	20.28	20.01	20.11	−0.02
35~	45.77	45.51	45.40	44.86	−0.67
60~	21.33	21.43	21.93	22.51	1.81
Proportion of severe disability	2.86	3.11	3.31	3.63	8.27
Birth rate (‰)	8.73	6.98	6.16	6.60	−8.90
Mortality rate (‰)	6.83	6.79	4.46	6.40	−2.14
Natural population growth rate (‰)	1.90	0.19	1.70	0.20	−52.78

### 4.1 Income forecast for the Shihezi City long-term care insurance fund

#### 4.1.1 The participation status of basic medical insurance in Shihezi City

The number of basic medical insurance participants in Shihezi City from 2014 to 2021 is shown in Table 2.

**Table 2:** The number of participants of basic medical insurance in Shihezi City from 2014 to 2021.

Year	Number of participants (person)
2014	559,881
2015	562,305
2016	563,669
2017	567,532
2018	577,474
2019	567,956
2020	569,100
2021	580,719

#### 4.1.2 Parameter confirmation of the exponential smoothing model

According to the principles based on initial value S0, if the sample size was <10, S0 took the average of the first 3 periods. Therefore, this study selected the initial value S0 as 561951.67. We selected all alternative smoothing coefficient alpha parameters between 0 and 1 and finally determined the alpha value which is calculated to be 0.30 by RMSE (Table 3).

**Table 3:** Parameter confirmation of the exponential smoothing model.

Code	Initial value S0	Smoothing coefficient alpha	Root mean square error value RMSE
1	561951.67	0.05	8792.58
2	561951.67	0.10	7810.37
3	561951.67	0.20	6713.35
4	561951.67	0.30*	6458.97
5	561951.67	0.40	6665.41
6	561951.67	0.50	7083.85
7	561951.67	0.60	7588.25
8	561951.67	0.70	8116.81
9	561951.67	0.80	8643.89
10	561951.67	0.90	9176.53
11	561951.67	0.95	9457.13

The smaller the root mean square error value (RMSE) is, the better the model prediction effect is.

#### 4.1.3 Prediction and fitting of the number of basic medical insurance participants in Shihezi City

The exponential smoothing model was used to predict and fit the number of basic medical insurance participants in Shihezi City, and the results are shown in Table 4. The relative error of the model in each year was less than 5%, and it had a high fitting accuracy. As forecasted, the number of the participants of basic medical insurance will be 577,867 in 2022. Additionally, according to the current fundraising standard of 50 yuan / person year, the income of long-term insurance fund will be 28.8934 million yuan in 2022.

**Table 4:** Prediction and fitting of the number of basic medical insurance participants in Shihezi City.

Year	Actual value	Predicted value	Residual	Relative error (%)
2014	559,881	559,881	0.00	0.00
2015	562,305	560,709	−1595.73	−0.28
2016	563,669	561,480	−2188.65	−0.39
2017	567,532	562,751	−4781.20	−0.84
2018	577,474	565,774	−11700.25	−2.03
2019	567,956	573,378.	5422.44	0.95
2020	569,100	571,763	2662.54	0.47
2021	580,719	571,315	−9404.44	−1.62
2022	–	577,867	–	–

If the relative error is less than 5%, the model’s fitting accuracy is considered high.

### 4.2 Shihezi City long-term care insurance fund compensation expenditure forecast

#### 4.2.1 Monthly compensation expenditure of Shihezi City long-term care insurance fund

The monthly compensation expenditure of Shihezi City long-term care insurance fund from August 2017 to September 2021 is shown in Table 5.

**Table 5:** Compensation expenditure of Shihezi City long-term care insurance fund from 2017 to 2021 (ten thousand Yuan).

Month	2017	2018	2019	2020	2021
1	–	74.68	139.69	178.89	202.63
2	–	25.67	135.82	172.03	196.62
3	–	62.39	140.53	178.77	211.35
4	–	71.69	146.16	180.11	214.50
5	–	80.68	147.72	182.25	217.94
6	–	79.57	148.90	195.23	220.26
7	–	78.50	154.89	189.93	221.67
8	4.90	92.08	155.79	192.00	230.87
9	16.62	116.84	161.26	189.66	232.92
10	24.04	131.30	164.64	195.73	–
11	22.86	131.39	173.85	199.09	–
12	32.72	133.86	176.85	203.16	–

#### 4.2.2 ARIMA model selection

An analysis of the original sequence diagram revealed that compensation expenditure of Shihezi long-term care insurance fund had an obvious tendency but no seasonality; therefore, a multiple non-seasonal ARIMA model was selected, namely ARIMA(p, d, q). The first order difference was used to make the sequence diagram stationary. After redrawing the sequence diagram, it was discovered that the sequence diagram after differential processing was clearly stationary; therefore, it was determined that *d* = 1.

We analyzed that p may take 0 or 1 and different ARIMA models of varying order were constructed. There were four alternative models, and a parameter test was performed to compare their fitting effects. The results are shown in Table 6.

**Table 6:** Order selecting of the ARIMA model for compensation expenditure of long-term care insurance fund.

Model	BIC	Ljung-Box Q Test		
		Statistic	*R^2^*	*p*
ARIMA(0,1,0)	5.252	15.384	0.004	0.635
ARIMA(1,1,0)	5.006	10.057	0.296	0.901
ARIMA(0,1,1)	4.895	4.772	0.370	0.998
ARIMA(1,1,1)	4.977	4.547	0.382	0.998

ARIMA(1,1,1), BIC = 4.977, R2 = 0.382, and the Ljung-Box Q test of this model showed that the statistic was 4.547, and there was no statistically significant difference (*p* = 0.998 > 0.05). The results indicated that the residual had a white noise, i.e., the residuals were randomly distributed. Furthermore, the model fully extracted the information from the original sequence diagram and was suitable for predicting the long-term insurance fund compensation expenditure.

#### 4.2.3 Forecast results of compensation expenditure of Shihezi City long-term care insurance fund

Table 7 shows the projected results of long-term insurance fund compensation expenditure from October 2021 to December 2022. According to the forecast, the total compensation expenditure of the long-term care insurance fund in 2022 totaled 28.4070 million yuan. The total expenditure of the 2022 long-term care insurance fund was estimated to be 30.7568 million yuan when combined with the compensation ratio of the 2021 long-term care insurance fund of 92.36%.

**Table 7:** Forecast of compensation expenditure of long-term care insurance fund (ten thousand Yuan).

Date	Predicted value	Predicted value 95% C.I.
October, 2021	232.20	212.33 ~ 252.08
November, 2021	235.47	214.92 ~ 256.02
December, 2021	238.27	216.27 ~ 260.27
January, 2022	237.27	214.06 ~ 260.47
February, 2022	236.46	212.08 ~ 260.83
March, 2022	235.86	210.37 ~ 261.35
April, 2022	235.48	208.92 ~ 262.04
May, 2022	235.31	207.72 ~ 262.90
June, 2022	235.35	206.78 ~ 263.93
July, 2022	235.61	206.08 ~ 265.14
August, 2022	236.08	205.62 ~ 266.54
September, 2022	236.76	205.41 ~ 268.12
October, 2022	237.66	205.43 ~ 269.89
November, 2022	238.77	205.68 ~ 271.85
December, 2022	240.09	206.18 ~ 274.00

#### 4.2.4 The fitting of the ARIMA model

The actual value of Shihezi City long-term care insurance fund compensation expenditure from October to December 2021 was fitted with the predicted value. All actual values were within 95% confidence intervals of the predicted values, and the relative errors were all within 3%. The prediction model had a good fitting effect. The results are as shown in Table 8.

**Table 8:** Results of the ARIMA model fitting (ten thousand Yuan).

Date	Actual value	Predicted value	95% C.I.	Relative error (%)
October, 2021	238.79	232.20	212.33 ~ 252.08	−2.76
November, 2021	237.14	235.47	214.92 ~ 256.02	−0.70
September, 2021	233.33	238.27	216.27 ~ 260.27	2.12

If the relative error is less than 5%, the model’s fitting accuracy is considered high.

#### 4.2.5 Forecast of long-term care insurance fund income and expenditure based on nursing fees

In 2021, the average cost of long-term care insurance for insured individuals in Shihezi City is 3,471.55 yuan/month/person. Since the current cost of home care cannot be statistically estimated, this study assumed that the average cost of home care insured was equal to the average cost of institutional nursing. According to the Pilot Opinions issued by the Ministry of Human Resources and Social Security, the fund will pay approximately 70% of the long-term care expenses of the insured. Based on the policy requirements, this study calculated that the average compensation nursing cost of the insureds of Shihezi City’ s long-term care insurance was 3471.55 yuan/month/person *70% = 2430.09 yuan/month/person.

The number of long-term care fund compensations (including institutional and home care) in 2021 was 29,339. Therefore, the compensation expenditure of long-term care fund in 2021 was estimated to be: 2430.09 yuan/month/person * 29339 person-time = 71296410.51Yuan.

The subsidy proportion of long-term insurance fund expenditure in 2021 was 92.36%, so it was estimated that the total expenditure of long-term care insurance fund in 2021 was 71296410.51 yuan/92.36% = 77194034.77 yuan.

The number of participants enrolled in long-term care insurance was 580,700. Therefore, the long-term care insurance *per capita* fund raising standard was estimated to be 77194034.77 yuan/ 580,700 person = 132.93 yuan/person/year.

## 5 Discussion

Several high-income countries, including Germany, the Netherlands, Luxembourg, Japan, and South Korea, utilize independent, specialized insurance plans for LTC services. In these countries, long-term care insurance is primarily funded through wage-based contributions tied to employment. Participation in LTC insurance is mandatory for the entire population or a subset of it in the aforementioned countries. With the exception of Japan, where only individuals aged 40 years and above can pay insurance premiums, working-age adults (or some countries’ retired populations) in these nations are required to pay mandatory insurance premiums for their LTC insurance (37). Consequently, coverage typically extends to all individuals in need of care.

In the first batch of pilot cities, the population base of Shihezi City was small, which facilitated the broad development of long-term insurance pilot work. Additionally, the increasing urbanization rate and gradually enhancing resident aggregation provided a better foundation for the implementation of long-term care insurance pilot work. Second, during the study, the aging rate of the Shihezi City population increased year by year, which was higher than the national average level during the same period. As the population ages, the demand for long-term care of residents also increases. According to National Guidelines, Shihezi City, in addition to covering the employees’ medical insurance, also extended coverage to residents’ medical insurance. This expansion broadened the scope of long-term care insurance coverage and achieved full population coverage. Since the pilot program initiation, the number of insured people under Shihezi City long-term care insurance had slowly increased, and by the end of 2021, 580,700 people were covered by long-term care insurance, with a 100% coverage rate. Although the coverage of the population of long-term care insurance in Shihezi City is broader, compared to developed countries abroad, the level of benefits is very limited (up to a maximum of only 750 yuan per month), which had an impact on application acceptance, fund raising, and the system’ s long-term sustainable operation.

Zhou Beixi’s research on the Yangtze River Delta region in China indicates that long-term care insurance in the area is mainly funded through fixed contributions based on residents’ disposable income. This approach makes it easily understood and accepted by residents (38). Similarly, in Shihezi City, funding is also structured in a fixed contribution format, with a funding standard of 50 yuan per person per year. This study showed that the first batch of pilot cities’ long-term care insurance funds were mainly from the medical insurance pooling fund (39). However, in the pilot implementation plan of Shihezi City long-term care insurance, the long-term care insurance fund was required to be raised through various channels, such as medical insurance pooling fund, personal contributions, welfare fund of lottery and financial subsidies. During the study, it was found that more than 95% of the long-term care insurance fund came from basic medical insurance fund, with the highest contribution reaching 100%, and the personal contributions, and other sources accounted for relatively low fundraising. On the other hand, during the same period, Shihezi City’s current basic medical insurance fund income was insufficient, and the fund with insufficient cumulative balance was at risk of running out at any time. Continuing to fund for long-term care insurance from basic medical insurance funds will increase the deficit medical insurance fund, which will be detrimental to the development of both the basic medical insurance system and the long-term care insurance system (40). Currently, the two main financing channels of social insurance (enterprises and government) face tremendous pressure in fundraising, which will not be conducive to the long-term economic development. Therefore, it is urgently necessary to establish sustainable financing mechanisms for multiple suppliers. In the long run, in order to ensure the sustainable development of the LTCI system, the government should establish a reasonable funding ratio for LTCI or use a combination of fund transfers and multi-source funding (41).

The Opinions on Expanding the Pilot Program issued by the Medical Insurance Bureau in 2020 suggested that the payment level from the insured’s fund was supposed to be generally controlled at approximately 70% of the nursing service fees that meet the requirements (42). This study calculated that, if the subsidy proportion of 70% was applied to the long-term care insurance fund in 2021 based on the nursing fees of long term care insurance beneficiaries, the *per capita* fund raising standard of the insured individuals should be 132.93 yuan/person/year. According to the document, long-term care insurance is primarily financed by companies and individuals. Companies and individuals should pay the same proportion of contributions in theory. Companies and individuals should each pay half, or 66.47 Yuan per person per year. This fund-raising standard accounts for 0.18% of residents’ *per capita* disposable income in 2020, and the payment pressure on residents is relatively light. It also implied that, if the individual payment obligation was implemented, the long-term care insurance fund can gradually reduce its reliance on the basic medical insurance fund and evolve into an independent insurance type. Without intervention, there is a risk that the income and expenditure of the LTCI fund will be unbalanced. By predicting the future development trend of the LTCI fund, it reflects the necessity and urgency of perfecting the financing system of LTCI and establishing a unified financing mechanism of LTCI. Priority should be given to meeting people’s healthcare needs first and then the social aspects of LTC needs. It should also be noted that, in almost all countries, funding for medical care is generally separate from funding for non-medical long-term care services (43).

Currently, most countries adopt diversified financing methods, with financing mechanisms taking into account the regional development and differences in household income. For example, the long-term care insurance system in Germany follows the principle of “triangular payment” with contributions from individuals, businesses, and government subsidies, where the state finances over a third, while individuals and businesses collectively cover the remaining costs (44). In Japan, 50% of long-term care insurance premiums are paid separately by the government and individuals, with a contribution ratio of 2: 1:1 between the central government, prefectures, and municipalities within public premiums (45). Therefore, Shihezi City should establish an LTCI financing mechanism composed of individuals, businesses, and the government, further increasing the government’s financial subsidies to urban and rural residents, implementing a payment system for retired employees, enhancing LTCI fund revenue, and improving the future fund’s payment capacity. Considering the characteristics of the “pay-as-you-go” system with annual payments, based on the principle of “expenditure determined by income and balancing revenues and expenditures,” a reasonable funding standard system should be constructed to maintain the stability and sustainability of the LTCI fund.

The data of this study come from the official data provided by relevant departments of the Shihezi City government, which ensures the accuracy of the data. By using the exponential smoothing model and the ARIMA model to forecast the income and expenditures of the old-age care insurance fund in 2022, and proposing the financing standard of the insured based on the forecast results, the research findings have more policy guidance significance and can provide a reference for promoting the sustainable development of the long-term care insurance. This study also has some limitations. First, the forecast assumes that the severe disability rate among residents of Shihezi City remains unchanged, which may impact the research results. However, according to surveys, there is not a significant variation in the severe disability rate among residents of Shihezi City. Therefore, the research results are unlikely to deviate significantly. Second, this study involves short-term forecasting and cannot reflect long-term changes in the financing of long-term care insurance in the city. Nonetheless, the long-term care insurance system in the city operates on a pay-as-you-go basis, where funding standards for each year are determined at the beginning of the year based on the previous year’s insurance expenditure. Therefore, employing short-term forecasting methods for the financing of long-term care insurance in the city is also beneficial.

In summary, the Shihezi City Mayor’s protection insurance system urgently needs to establish a multi-channel financing mechanism of mutual assistance and shared responsibility. It can expand the sources of welfare supply by integrating fund sources, such as individual payment, welfare and sports lottery income, and social donation, and gradually establish multi-channel financing of risk sharing (46). Second, the corresponding fund needs of basic nursing services should be scientifically calculated; the annual total amount of regional financing should be reasonably determined; the obligation of individual payment should be emphasized; the individual financing standard should be appropriately raised; the pressure on the medical insurance fund should be alleviated; and the stable operation of the long-term care insurance fund should be guaranteed (47).

## Data availability statement

The datasets presented in this study can be found in online repositories. The names of the repository/repositories and accession number(s) can be found in the article/supplementary material.

## Author contributions

XY: Project administration, Writing – original draft. PZ: Data curation, Formal analysis, Writing – review & editing. HX: Funding acquisition, Project administration, Resources, Writing – review & editing.
